# MPRAdecoder: Processing of the Raw MPRA Data With *a priori* Unknown Sequences of the Region of Interest and Associated Barcodes

**DOI:** 10.3389/fgene.2021.618189

**Published:** 2021-05-11

**Authors:** Anna E. Letiagina, Evgeniya S. Omelina, Anton V. Ivankin, Alexey V. Pindyurin

**Affiliations:** ^1^Institute of Molecular and Cellular Biology of the Siberian Branch of the Russian Academy of Sciences, Novosibirsk, Russia; ^2^Faculty of Natural Sciences, Novosibirsk State University, Novosibirsk, Russia

**Keywords:** massively parallel reporter assay, MPRA, reporter constructs, region of interest, barcodes, next-generation sequencing, NGS data processing, pipeline

## Abstract

Massively parallel reporter assays (MPRAs) enable high-throughput functional evaluation of numerous DNA regulatory elements and/or their mutant variants. The assays are based on the construction of reporter plasmid libraries containing two variable parts, a region of interest (ROI) and a barcode (BC), located outside and within the transcription unit, respectively. Importantly, each plasmid molecule in a such a highly diverse library is characterized by a unique BC–ROI association. The reporter constructs are delivered to target cells and expression of BCs at the transcript level is assayed by RT-PCR followed by next-generation sequencing (NGS). The obtained values are normalized to the abundance of BCs in the plasmid DNA sample. Altogether, this allows evaluating the regulatory potential of the associated ROI sequences. However, depending on the MPRA library construction design, the BC and ROI sequences as well as their associations can be *a priori* unknown. In such a case, the BC and ROI sequences, their possible mutant variants, and unambiguous BC–ROI associations have to be identified, whereas all uncertain cases have to be excluded from the analysis. Besides the preparation of additional “mapping” samples for NGS, this also requires specific bioinformatics tools. Here, we present a pipeline for processing raw MPRA data obtained by NGS for reporter construct libraries with *a priori* unknown sequences of BCs and ROIs. The pipeline robustly identifies unambiguous (so-called genuine) BCs and ROIs associated with them, calculates the normalized expression level for each BC and the averaged values for each ROI, and provides a graphical visualization of the processed data.

## Introduction

Although numerous regulatory elements have been identified in eukaryotic genomes (Narlikar and Ovcharenko, 2009; Taher et al., 2011; Kellis et al., 2014), so far there is no complete understanding of why these elements are active in specific cell types and at specific levels. Accordingly, the effect of a particular mutation within a regulatory element can be hardly predicted, especially for a particular cell type (1000 Genomes Project Consortium et al., 2015; Albert and Kruglyak, 2015; Rojano et al., 2019). The recent development of massively parallel reporter assays (MPRAs) allows high-throughput functional characterization of native transcriptional regulatory elements (first of all, enhancers and promoters) as well as their mutant variants (reviewed in Haberle and Lenhard, 2012; Inoue and Ahituv, 2015; Trauernicht et al., 2020; Mulvey et al., 2021). In an MPRA, regions of interests (ROIs), e.g., putative enhancers or promoters, together with unique barcodes (BCs) are assembled into reporter constructs to obtain MPRA plasmid libraries that consist of thousands or even millions of individual molecules (Kheradpour et al., 2013; Kwasnieski et al., 2014; van Arensbergen et al., 2019). Specific MPRA libraries can also be packaged in lentiviruses to deliver reporter constructs into the target genome (O’Connell et al., 2016; Inoue et al., 2017; Maricque et al., 2017; Gordon et al., 2020).

**FIGURE 1:**
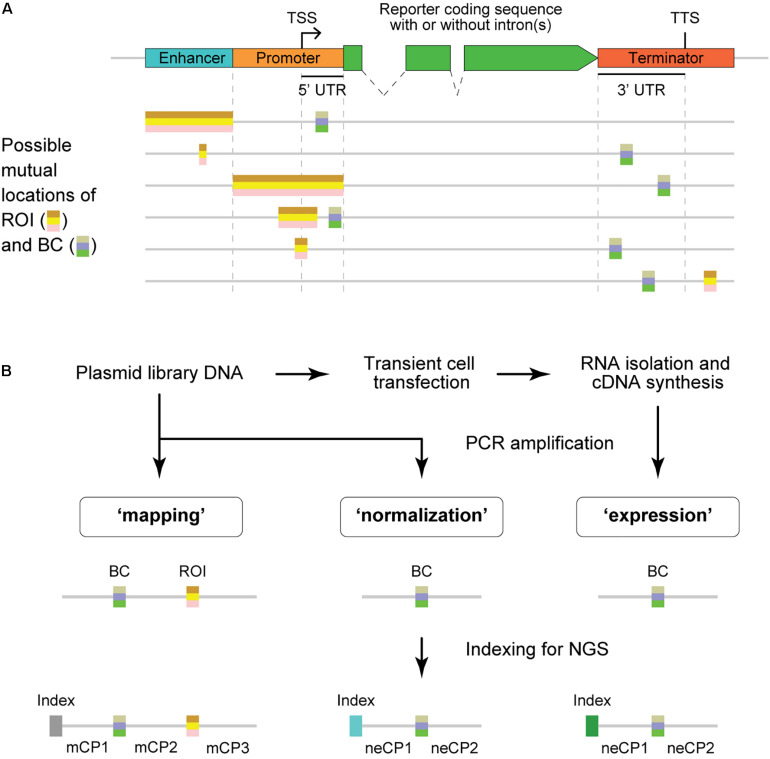
Structure of MPRA constructs and samples. **(A)** Schematic representation of typical MPRA reporter constructs with two variable regions, the region of interest (ROI) and barcode (BC). An enhancer element may be missing in particular MPRA constructs. The ROI is usually located outside the coding sequence, whereas the BC must be within the transcribed unit to ensure measurements of the ROI influence on the reporter expression level. TSS, transcription start site; TTS, transcription termination site; UTR, untranslated region. **(B)** Schematic showing the experimental steps involved in the preparation of MPRA samples and their structures. Note that the normalization and expression samples have identical structures; the mapping sample is required only for MPRA libraries in which BC–ROI associations are not predetermined. mCP, constant part present in the sequence of the mapping sample; neCP, constant part present in the sequence of the normalization/expression sample.

From the structural point of view, BCs are always placed within the transcription unit [usually in the 5′ or 3′ untranslated region (UTR)], whereas ROIs are typically outside this unit (Figure 1A). As a result, the BC sequences are present in the reporter mRNA molecules and, thus, allow quantitative evaluation of the regulatory effects caused by their *cis*-paired ROI variants using next-generation sequencing (NGS) (Figure 1B and Supplementary Figure 1). For that, cells of interest are transfected by an MPRA plasmid library or transduced by a lentiviral MPRA library, and subsequently, transcriptional activity levels of barcoded reporters are assessed on episomal plasmids and/or after stable integration of the constructs at random or specific genomic loci (Melnikov et al., 2012; Sharon et al., 2012; Kheradpour et al., 2013; White et al., 2013; O’Connell et al., 2016; Tewhey et al., 2016; Ulirsch et al., 2016; Maricque et al., 2017; Inoue et al., 2019). More specifically, the “expression” and “normalization” samples are prepared by PCR amplification of the BC sequences from cDNA synthesized on total RNA isolated from the transfected/transduced cells and the plasmid DNA used to transfect cells or total DNA isolated from the transduced cells, respectively. These samples are subjected to NGS to determine the normalized expression level of each BC, which is calculated as the ratio between the BC abundance in the expression and normalization samples.

It should be noted that ROIs can be either (i) preselected native, mutant, and/or synthetic sequences (e.g., minimal core elements of enhancers and promoters) usually of the same length (Melnikov et al., 2012; Sharon et al., 2012; Kheradpour et al., 2013; Smith et al., 2013) or (ii) somehow experimentally enriched genomic fragments, random genomic fragments, or synthetic sequences of varying length (Mogno et al., 2013; Verfaillie et al., 2016; van Arensbergen et al., 2017). In particular cases, the ROI can be just a fixed segment within the cloned regulatory element (Patwardhan et al., 2009; Vvedenskaya et al., 2015; Omelina et al., 2019). On the other hand, BCs are most frequently sequences of fixed length between 9 and 20 nucleotides (nts) (Kwasnieski et al., 2012; Melnikov et al., 2012; Patwardhan et al., 2012; Mogno et al., 2013; Verfaillie et al., 2016).

Depending on the MPRA library design, the ROI and BC sequences as well as their associations can be either *a priori* known or not. Completely predetermined MPRA libraries are generated by using sequences synthesized on custom high-density DNA microarrays (Patwardhan et al., 2009; Melnikov et al., 2012; Sharon et al., 2012; Kwasnieski et al., 2014). MPRA libraries with unknown sequences of ROIs and BCs are made by cloning randomly sheared genomic fragments or pooled synthetic DNA fragments or by PCR-mediated mutagenesis and/or by cloning oligonucleotides containing randomized stretches of nucleotides (Patwardhan et al., 2012; Mogno et al., 2013; Vvedenskaya et al., 2015; Verfaillie et al., 2016; van Arensbergen et al., 2017; Kircher et al., 2019; Omelina et al., 2019). In some cases, the ROI sequences are predetermined although associated BCs are not known in advance (Smith et al., 2013; O’Connell et al., 2016; Tewhey et al., 2016; Grossman et al., 2017; Gordon et al., 2020). For the libraries that are not completely predetermined, there is a need to identify cloned ROI and/or BC sequences as well as their associations. Hereafter, the procedure of finding unique BC–ROI associations is referred to as “mapping” by analogy with the thousands of reporters integrated in parallel (TRIP) experiments (Akhtar et al., 2013, 2014). The mapping is typically done by PCR amplification of BC–ROI regions of MPRA constructs followed by Illumina NGS (Patwardhan et al., 2012; Mogno et al., 2013; Tewhey et al., 2016; Omelina et al., 2019). Importantly, associations of the same BC with different ROI sequences are excluded from the further analysis although the association of the same ROI with different BCs allows revealing and excluding the possible influence of particular BC sequences on the measurements.

The MPRAdecoder pipeline described in this study was developed for the processing of NGS data generated for MPRA libraries with *a priori* unknown sequences of ROIs and BCs, for example, those cloned by the usage of oligonucleotides with randomized stretches of nucleotides. The pipeline (i) robustly identifies unambiguous (hereafter genuine) BCs and their mutant variants as well as associated ROIs, (ii) calculates the normalized expression level for each genuine BC and the averaged values for each ROI, and (iii) provides a graphical visualization of the processed data. The functionality of the pipeline was demonstrated using a data set obtained for an MPRA library designed to study the effects of sequence variations located at a certain distance downstream of the transcription termination site (TTS) of the *eGFP* reporter on its expression at the transcription level.

## Materials and Methods

### Preparation of the MPRA Mapping, Expression, and Normalization Samples and Illumina NGS

The MPRA plasmid library, in which random-sequence BC and ROI are separated by an 83-nt fixed-sequence region and located, respectively, in 3′ UTR and downstream of the TTS of the *eGFP* reporter, was generated earlier (Omelina et al., 2019). The wild-type and mutant deltaC (Boldyreva et al., 2021) reporter plasmids carrying specific 20-nt BCs were constructed by standard molecular cloning procedures and verified by sequencing. An equimolar pool of two such wild-type and two deltaC mutant plasmids was mixed in a 1:99 molar ratio with the MPRA plasmid library. Immortalized human embryonic kidney (HEK293T) cells were obtained from ATCC (United States) and were maintained and transfected as described previously (Boldyreva et al., 2021).

The mapping samples were prepared according to a previously reported two-round conventional PCR procedure that prevents the formation of chimeric products (Omelina et al., 2019). Briefly, primers specific to the ends of fixed sequences mCP1 and mCP3 (Figure 1B and Table 1) were used, and a specific, custom-designed 8-nt index along with other sequences necessary for Illumina NGS was introduced in the PCR products of each sample replicate. The normalization samples were obtained in the same way, using primers specific to the ends of fixed sequences neCP1 and neCP2 (Figure 1B and Table 1) and 2.5 ng of the plasmid library as a template. To prepare expression samples, BCs were amplified as specified above but using 1/20 of cDNA prepared from the transfected cells as described earlier (Boldyreva et al., 2021) as a template. Phusion High-Fidelity DNA Polymerase (Thermo Fisher Scientific) was used for all amplification reactions. All obtained PCR products were purified on spin columns, mixed together, and sequenced on an Illumina MiSeq instrument as 151-nt single-end reads. Notice that the read length was shorter than the amplified plasmid fragments for all samples. Therefore, there was no need to remove Illumina adapter sequences from the reads. Finally, to prepare an example data set, a representative subset of the reads was randomly selected from the obtained fastq file. A copy of this subset was demultiplexed using Cutadapt (Martin, 2011).

**TABLE 1:** Specific features of the example MPRA data set.

**Part^a^**	**Length, nts**	**Strand^b^**	**Sequence**	**Note**
**“Mapping” sample**				
index	8	Plus	AGCGAGCT, CTGCACGT	Fixed
mCP1	17	Plus	GACACTCGAGGATCGAG	Fixed
BC	18^c^	Plus	(N)_18_	Random
mCP2	83	Plus	GAGTTGTGGCCGGCCCTTGTGACTGGGAAAACCCTGGCGTAAAT AAAATACGAAATGACTAGTCATGCGTCAATTTTACGCAT	Fixed
ROI	8	Plus	(N)_8_	Random
mCP3	17^d^	Plus	TTAACGTACGTCACAATATGATTATCTTTCTAGGG^e^	Fixed
**“Normalization” and “Expression” samples**				
index	8	Plus	CCTATGGT, AACGTCGT, ACAATTCG, TACTTGTC	Fixed
neCP1	39	Minus	CGCCAGGGTTTTCCCAGTCACAAGGGCCGGCCACAACTC	Fixed
BC	18^c^	Minus	(N)_18_	Random
neCP2	86^d^	Minus	CTCGATCCTCGAGTGTCACCTAAATCGTATGCGGCCG CGAATTCTTACTTGTACAGCTCGTCCATGCCGAGAGTGATCCCGGCGGC GGTCACGAACTCCAGCAGGAC^e^	Fixed

*^a^In the order of presence in the sample (starting from immediately after the Illumina forward sequencing primer). ^b^Orientation relative to the plasmid reporter construct, except the index introduced during PCR amplification. ^c^The length of the BCs present in the spiked-in reference constructs was 20 nts (see the text for details). ^d^The expected length of the fragment of the component in 151-nt single-end reads; the length is shorter by 2 nts for the reads containing reference 20-nt BCs. ^e^The complete sequence of the component in the PCR amplified sample is shown; the fragment expected in 151-nt single-end reads is underlined.*

### Pipeline Code and Documentation Availability

The MPRAdecoder pipeline source code written in Python, the example data set, and the corresponding expected outputs as well as detailed documentation are publicly available on GitHub repository^1^.

### Hardware and Software Requirements

The MPRAdecoder installation and analyses were performed on a computer with an Intel^®^ Core^TM^ i7-3770 processor, 31.4 Gb RAM, Linux Ubuntu 14.04 64-bit system, and Python version 3.8.6.

## Results

### Overview of the MPRAdecoder Pipeline

**FIGURE 2:**
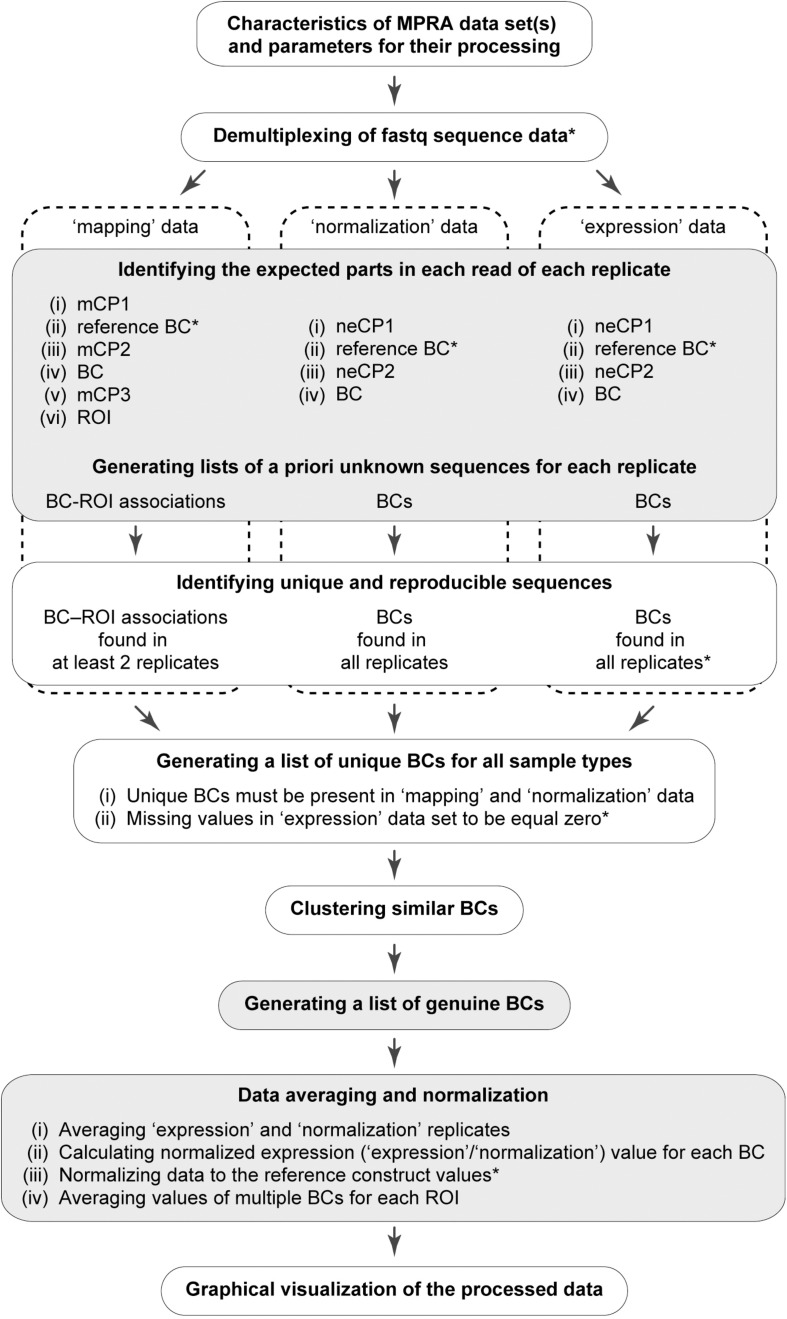
Schematic overview of the MPRAdecoder pipeline. The optional steps are marked by an asterisk. The steps at which the pipeline saves intermediate or final results are marked by gray.

A workflow of the MPRAdecoder pipeline is shown in Figure 2. Briefly, after providing details of a particular MPRA data set to be analyzed, the pipeline parses the input fastq file(s) and demultiplexes them if required. Next, all expected parts of the mapping, normalization, and expression reads are detected, particularly the sequences of BCs and ROIs. Then, a list of BCs common for all samples is generated with the assumption that some BCs have zero counts in the expression data. After that, genuine BCs and their mutant variants as well as associated ROIs are identified. Finally, the data are averaged over expression and normalization replicates, normalized, and averaged over ROIs, and the results are visualized in different plots. Below, these steps are described in more detail with the help of the example MPRA data set.

### Characteristics of the Example Data Set

To demonstrate the capabilities of the MPRAdecoder pipeline, we used a data set consisting of two biological replicates of mapping, normalization, and expression samples obtained using an MPRA library, in which the BC and ROI (both cloned by using oligonucleotides containing fully randomized sequences) are located in 3′ UTR and downstream of TTS, respectively (the option is shown at the bottom of Figure 1A), being separated by 83 nts of fixed sequence (Omelina et al., 2019). The samples were sequenced as 151-nt single-end reads on the Illumina MiSeq platform and were indexed with custom-designed 8-nt sequences located at the beginning of the reads (Figure 1B). Important features of the data set are listed in Table 1. Note that the BC sequences were in forward and reverse-complement orientations in the mapping and normalization/expression samples, respectively. In addition, about 1% of the reads in each sample contained four unique 20-nt BCs associated with spiked-in reference constructs; the TTCCAAGTGCAGGTTAGGCG and TGTGTACGGCTTGCTCTCAA sequences tagged the wild-type construct, whereas GAGCCCGGATCCACTCCAAG and TGTCACGTCAGCTAACCCAC sequences marked the deltaC mutant construct that is characterized by a higher expression level than the wild-type one (Boldyreva et al., 2021). The substantially longer length of the BC (18 nts) compared to the ROI (8 nts) ensures that each ROI is associated with multiple different BCs in a representative large plasmid library. This allows controlling the potential influence of individual BC sequences on the studied phenomenon.

### Specifying Characteristics of an MPRA Data Set to Be Analyzed

The information on the input MPRA data set is provided in the two complementary forms. First, most details, such as (i) names and lengths of all expected parts in the mapping and normalization/expression reads for each MPRA library (including indexes), (ii) sequences of the predetermined parts (including indexes and optional reference BCs), (iii) relative orientation of BC sequences in mapping and normalization/expression reads, (iv) a maximum allowed error rate and the Phred quality score threshold for different parts, (v) a minimum number of read counts required for a BC and a BC–ROI association, and (vi) settings for identification of genuine BCs and associated ROIs, are specified in the configuration file. A detailed description of this file is available on the GitHub page of this project. Second, a user has to manually input the following details in the command prompt: (vii) names of the appropriate fastq file(s) and their locations as well as a location for output files, (viii) a number of replicates of each sample for each MPRA library, (ix) names of indexes used for sample multiplexing and (x) information on whether the fastq file(s) should be demultiplexed by the pipeline.

### MPRA Data Demultiplexing by Pairwise Sequence Alignment

The pipeline is able to process either fastq files that are already demultiplexed, for example, by the Illumina software, or fastq files containing custom-designed index sequences at the beginning of the reads. In the latter case, detection of a predetermined index sequence in each read is performed using a pairwise sequence alignment tool from Biopython (Cock et al., 2009). For that, all index sequences specified in the configuration file are aligned, one by one, against the beginning of a read. The following alignment scoring system is used: +1 for a match, 0 for a mismatch, and –1 for an indel. If the maximum alignment score is higher than or equal to the threshold value (calculated as the index length - the maximum allowed error rate + 1 for each insertion) and the Phred quality score for each base (Cock et al., 2010) is higher than a threshold (equal to 10 for the example data set), the corresponding index sequence is considered to be identified; otherwise, the read is discarded. To generate the example data set, 8-nt index sequences differing from each other by at least 2 nts were used as suggested for the short (5–10 nts) predefined BCs (Patwardhan et al., 2009; Sharon et al., 2012). At the same time, the maximum allowed error rate was set to ∼10% based on our experience with PCR-amplification and subsequent NGS of predetermined sequences under experimental conditions identical to those used in this study (including the quality of oligonucleotide primers). Together, these factors ensure that one allowed single-base mutation (substitution, deletion, or insertion) in the index sequence cannot lead to an error in its identification. At the end, the reads are divided into an appropriate number of groups based on the detected indexes.

### Identification of the BC and ROI Sequences in the Reads

Detection of the mCP1, mCP2, mCP3, neCP1, neCP2 (Figure 1B and Table 1), and reference BC sequences in the reads is performed for each replicate of each sample by using the pairwise sequence alignment approach described above for the index, taking into account location(s) of the preceding part(s), which can be already identified (e.g., the mCP1/neCP1) or just estimated (e.g., the BC). Sequences of BCs and ROIs are defined as spacers between the appropriate constant parts. By default, the Phred quality scores are ignored for the mCP1, mCP2, mCP3, neCP1, and neCP2 sequences. For the BCs (including the reference ones) and ROIs, the quality score for each base should be higher than a threshold (e.g., set to 10 for the example data set); otherwise, reads are discarded from the downstream analysis. More specifically, in the case of the mapping reads, the process includes the following sequential steps. First, the mCP1 sequence is detected. Second, if sequences of the reference BCs are specified in the configuration file, the reads with such BCs are identified and excluded from the subsequent structural analysis. This is done because the functional sequences (e.g., wild-type or deltaC in the example data set) associated with the reference BCs might be located outside the ROI (e.g., within the mCP2 sequence as in the example data set). Third, the mCP2 sequence is detected, and the sequence between mCP1 and mCP2 is recognized as the BC if its length is within the range set in the configuration file (e.g., ≥16 and ≤20 nts for the example data set). Fourth, the mCP3 sequence is identified, and the sequence between mCP2 and mCP3 is recognized as the ROI if its length is within the range defined in the configuration file (e.g., ≥7 and ≤9 nts for the example data set). In the case of the normalization and expression reads, the last step is omitted. Lastly, if the ROI and/or BC sequences are in reverse-complement orientations in the mapping or normalization/expression samples (this is specified in the configuration file), they are converted to their forward counterparts.

### Data Filtering and Generation of a List of Unique BCs

At the next step, the number of supporting reads for each BC (with a random or reference sequence) is counted for each replicate of all samples. Then, these numbers are divided by the total number of effective reads (i.e., those that passed all filters described above) in a replicate and multiplied by 1 × 10^6^ to calculate the reads per million (RPM) values. After that, unique BC–ROI associations and BCs are assessed for reproducibility and robustness. Although preliminary results can be obtained using single replicates of the mapping, normalization, and expression samples, at least two replicates of each sample are strongly recommended. Under such conditions, only the BC–ROI associations that are revealed with at least *m* raw read counts (e.g., one for the example data set) in at least two out of any available number of replicates of the mapping data are retained for further analysis. Also, only the BCs with *n* raw read counts (e.g., three for the example data set) in each replicate of the normalization data are kept. For the expression data, the threshold read count *e* is set by default to zero, as some BCs might be present with very low frequency or even completely absent in the reporter transcripts due to the properties of particular ROI sequences. The threshold values *m*, *n*, and *e* are arbitrarily set in the configuration file. Finally, a list of BCs that are common for all samples is generated considering that some BCs might have zero counts in some or all replicates of the expression data.

### Identification of Genuine BCs

Oligonucleotides with a totally randomized part (characterized by an equal representation of all four nucleotides at each position) of 15–20 nts in length can ensure cloning of ∼1 × 10^9^ to 1 × 10^12^ unique BCs, some of which might be different from each other just at one position. However, in practice, the size of a typical MPRA plasmid library is significantly less (by orders of magnitude) than the theoretical values. Nevertheless, in MPRA data sets, BCs with similar sequences do appear, partly due to errors introduced during PCR amplification and NGS steps. Thus, there is a need to find similar BC sequences, group them, and identify the genuine BCs in each such group (referred to below as a cluster). Two BC sequences are considered to be similar if they differ at no more than *s* positions (by substitutions, deletions, and/or insertions), where *s* is equal to the maximum allowed error rate for this part. By default, up to two mismatches

are allowed for BCs of the example data set, as suggested previously (Akhtar et al., 2013).

**FIGURE 3:**
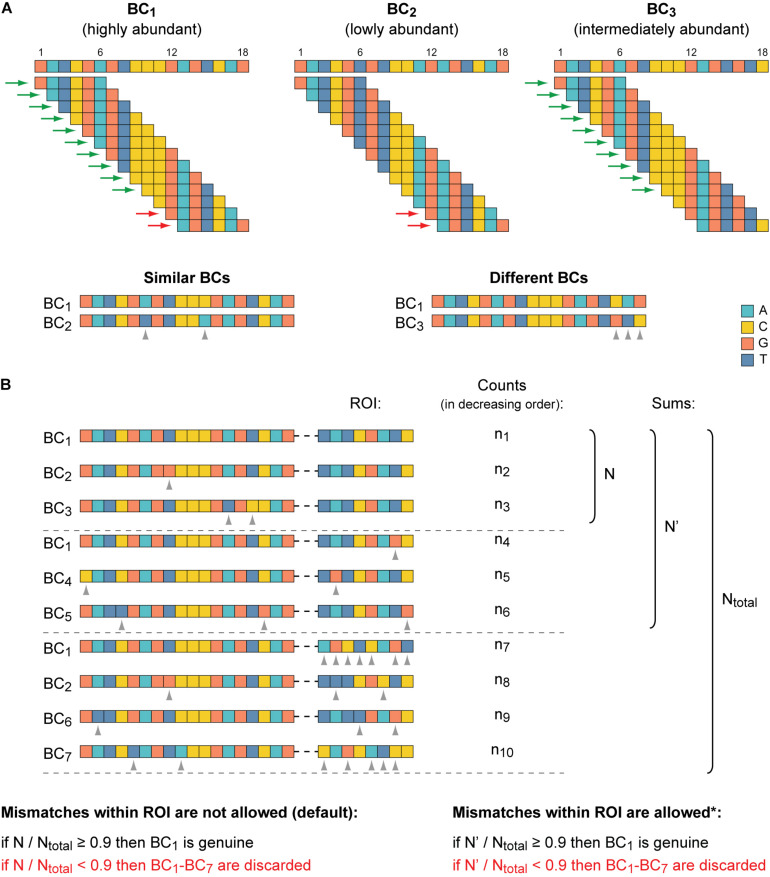
Identification of genuine BCs, their mutant variants, and associated ROIs. **(A)** The clustering of similar BC sequences is achieved by their decomposition into overlapping k-mers and by the subsequent pairwise alignment of BCs that share identical k-mers. At the top, three BCs are shown as an example. K-mers (6-mers) shared by BC_1_ and BC_2_ and by BC_1_ and BC_3_ are indicated by red and green arrows, respectively. At the bottom, the pairwise sequence alignment of the candidate similar BC sequences is depicted. The BC_1_ and BC_2_ are recognized to be similar because their sequences differ from each other only at two positions (≤the maximum allowed error rate). BC_1_ and BC_3_ are considered to be different because their sequences differ from each other at three positions (>the maximum allowed error rate) even though these BCs share more common k-mers than the BC_1_ and BC_2_. **(B)** Identification of genuine BCs. One cluster of seven similar BCs along with the associated ROI sequences is shown as an example. BC_1_ is the most abundant BC (as in **A**) and the ROI sequence, which is associated with it most frequently (n_1_ > n_4_ and n_1_ > n_7_), is considered as the putative ROI for the cluster. By default, if the putative ROI is supported by at least 90% of normalized read counts calculated for all ROI sequences found in the cluster, the BC_1_ becomes genuine. Otherwise, the entire cluster is excluded from the subsequent analysis. Optionally (indicated by an asterisk), if mismatches within the ROI are permitted (e.g., a difference at one position could be allowed for the example data set), then normalized read counts for the putative ROI and its allowed mutants are summed. Notice that differences between the ROI sequences associated with similar BCs should be allowed with caution, especially for very short ROIs. Dashed horizontal lines separate different groups of ROIs: the putative sequence, its allowed mutants, and all other sequences. Gray arrowheads denote mismatches in both panels.

Because identification of similar BCs by the means of alignment approaches is rather time-consuming, especially for thousands or even millions of sequences to compare (Song et al., 2014; Zielezinski et al., 2017), the MPRAdecoder pipeline first preselects candidate BCs for their subsequent pairwise sequence alignment (Figure 3A). The preselection is achieved by decomposing all unique BC sequences into overlapping k-mers and then revealing BCs that share identical k-mers (Haubold, 2014; Zielezinski et al., 2017). The length of k-mers (e.g., six for the example data set) is calculated as the BC length/(*s*+ 1) rounded down to the nearest whole number. Next, BCs sharing each particular k-mer are directly compared by using the pairwise sequence alignment (see above), taking into account their normalized read counts (RPM values). Then, similar BCs are grouped into clusters, and a number of quality control steps are applied to ensure the absence of overlap between the clusters (ambiguous cases are removed).

After that, for each cluster, it is verified whether the most abundant ROI associated with the most abundant BC is supported by the majority of normalized read counts obtained for all ROI sequences present in a cluster (Figure 3B). As a default setting, an arbitrary cutoff at ≥ 0.9 (specified in the configuration file) is used, similar to earlier studies (Akhtar et al., 2013; Mogno et al., 2013). If the criterion is not satisfied, probably due to associations of the same BC with different ROIs during the cloning by a chance or formation of chimeric molecules during PCR amplification of the mapping samples (Omelina et al., 2019), the entire cluster is excluded from the downstream analysis. If the criterion is satisfied, the most abundant BC and all other BCs are recognized as genuine and its mutant variants, respectively (the appropriate information is saved in a tab-delimited text file), and the RPM values of all BCs in such cluster are summed for each replicate of each sample. Eventually, all genuine BC sequences are different from each other by at least *s* + 1 position(s) (e.g., three for the example data set).

### Data Normalization and Visualization

Once genuine BCs are identified, their RPM values in the normalization and expression replicates are averaged. Next, for each genuine BC, the normalized expression value is calculated as a ratio between its expression and normalization RPM values. Then, if reference constructs were spiked in the plasmid library, the pipeline can further normalize data by dividing them by the value obtained for one of these references (specified in the configuration file; e.g., for the wild-type construct in the case of the example data set). After that, values obtained with different genuine BCs but for the same ROI sequence are averaged. The raw and normalized read counts per unique BC–ROI association for each replicate of the mapping samples and per unique BC for each replicate of the expression and normalization samples, the RPM values averaged over these replicates as well as the ultimate expression values obtained for genuine BCs after each step of the data normalization and averaging are saved as tab-delimited text files. Also, the important details of data processing are reported in additional files. Among them are the numbers of allowed mismatches in the expected parts of the reads; the list of input fastq files used for a run; and statistics on (i) total and effective read counts per fastq file, (ii) numbers of unique and genuine BCs, and (iii) numbers of genuine BCs per ROI.

**FIGURE 4:**
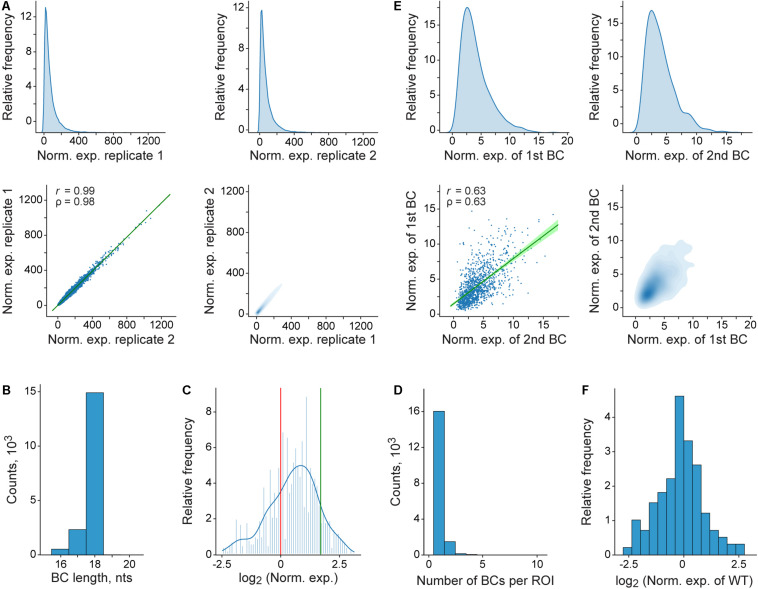
Representative plots generated by the pipeline. **(A)** Distribution and correlation of normalized expression values for the entire MPRA library. At the top, density plots of normalized expression values obtained for genuine BCs in each of the two replicates are shown. At the bottom, the correlation of these values between the replicates is visualized as regular (left) and density (right) scatterplots. In the left scatterplot, the regression line and 99% confidence intervals are shown in green; *r* and ρ denote Pearson’s correlation coefficient and Spearman’s rank correlation coefficient, respectively. **(B)** Genuine BC length distribution for the entire MPRA library. **(C)** Histogram and a kernel density estimation of normalized expression values for the entire MPRA library that were averaged over replicates and normalized to the wild-type reference construct. The expression levels of the wild-type and the deltaC mutant references are indicated by red and green vertical lines, respectively. **(D)** Distribution of the genuine BC number per ROI for the entire MPRA library. **(E)** Effect of the BC sequences on normalized expression values as estimated by using a subset of the ROIs, each associated with more than one BC. For each such ROI, only two different BCs, which are randomly assigned to groups “1st BC” and “2nd BC” are used for the comparison (for the ROIs associated with three or more BCs, only two of them are randomly sampled). At the top, density plots of normalized expression values obtained for BCs from the groups “1st BC” and “2nd BC” are shown. At the bottom, the correlation of these values between the groups is visualized as regular (left) and density (right) scatterplots. The rest of the description of the left scatterplot is as in **(A)**. **(F)** Distribution of normalized expression values of genuine BCs, each associated with the ROI of the wild-type (WT) sequence. For **(A–F)**, it is worthwhile noting that the plots shown were generated by using the entire fastq file obtained (see section “Materials and Methods”), from which the example data set was randomly sampled.

Finally, the pipeline generates a number of plots to help evaluate data quality and interpret the results (Figure 4). In particular, the reproducibility of the measurements between the replicates of the expression and normalization samples, the potential influence of the BC sequences on the measurements, and the sequence peculiarities of the ROIs with different properties are visualized.

### Performance of the Pipeline

The pipeline can process 1 million reads of a non-demultiplexed fastq file in ∼20 min using the hardware and software specified in Materials and Methods. For larger data sets, the processing time can be estimated by assuming a linear dependence on the read number.

## Discussion

MPRAs are becoming widely used as an effective tool to assess functionality of *cis*-regulatory DNA elements in a high-throughput manner (Ernst et al., 2016; Rabani et al., 2017; Mattioli et al., 2019; Shigaki et al., 2019; Choi et al., 2020; Davis et al., 2020; Ireland et al., 2020; King et al., 2020; Klein et al., 2020; Morgan et al., 2020; Renganaath et al., 2020). In addition, several modifications to the approach have been described that broaden its applicability (Rosenberg et al., 2015; Shen et al., 2016; Safra et al., 2017). Accordingly, to simplify the design of the MPRA experiments as well as to analyze their results, a number of bioinformatics pipelines have been developed, the majority of which were, however, so far validated primarily for studies with predetermined sequences of both ROIs and BCs or, at least, ROIs (Georgakopoulos-Soares et al., 2017; Ghazi et al., 2018; Kalita et al., 2018; Ashuach et al., 2019; Myint et al., 2019; Niroula et al., 2019; Gordon et al., 2020; Qiao et al., 2020; Yang et al., 2021).

The MPRAdecoder pipeline is primarily intended for the processing of data obtained for MPRA libraries generated using oligonucleotides with randomized stretches of nucleotides for cloning the ROI and BC sequences. Such libraries are most suitable for the investigation of the properties of all possible sequence variants within a certain small region of a regulatory element. Considering the current capabilities of NGS as well as the necessity for several different BCs per ROI, the length of the region that can be subjected to saturation mutagenesis is in the range of 8–10 nts. The need for multiple BCs per ROI is dictated by the following two main factors. First, the BC sequences themselves might influence the measurements performed (Ernst et al., 2016; Ulirsch et al., 2016; Figure 4F), most probably due to occasional occurrence of binding sites for specific DNA- or RNA-binding proteins or microRNA in them. Therefore, in order to identify and exclude such cases, it is necessary to analyze each ROI sequence in combination with different BCs. Second, mutations may appear in both the ROI and BC sequences due to errors in PCR amplification and NGS although the frequency of such events was previously estimated to be relatively low (the error rate per nt ≤ 0.3%) (Pfeiffer et al., 2018; Ma et al., 2019). At the same time, all possible variants of the short ROI sequence are expected to be present in a high-quality MPRA library, making identification of mutant ROI variants in the reads practically impossible. However, the use of multiple BCs for each ROI allows detecting outliers, which can be, in particular, caused by mutated ROI sequences, and excluding them from the analysis.

Multiple BCs per ROI can be simply ensured by a longer sequence of the BCs compared to the ROIs (e.g., 18 and 8 nts, respectively, in the example MPRA library). In addition, such design allows excluding as much as possible mutant or just very similar BC sequences from the analysis. Namely, only such BCs (referred to as genuine) (Akhtar et al., 2013; Omelina et al., 2019) are used, which sequences differ from each other by at least a certain number of nts. For example, when predefined BCs up to 20 nts in length are used, the difference between each pair of them of at least at two to three positions is typically set (Patwardhan et al., 2009; Sharon et al., 2012). For BCs with random sequences of 16 nts in length, the minimum difference at three positions also provides reliable measurements (Akhtar et al., 2013, 2014). In our case, we linked the allowed error rate in the BC sequences (as well as in all other parts of the reads, except for the ROI, in which we do not allow errors by default) with the experimentally determined error rate detected for fixed sequences amplified and sequenced in same conditions. Note that with the ROI length of 8 nts, a total of 4^8^ = 65,536 sequence variants are possible, whereas the BC length of 18 nts provides 4^18^ = 68,719,476,736 variants. Of the latter, obviously, not all can be genuine BCs (satisfy the Levenshtein distance ≥ 3) (Faircloth and Glenn, 2012; Hawkins et al., 2018), but nevertheless, each ROI can be associated with more than enough number of different BCs.

The use of oligonucleotides with randomized stretches of nucleotides to clone the ROIs and BCs as well as regular primers to amplify the mapping, normalization, and expression samples means that the following considerations should be taken into account during the processing of raw MPRA data. First, although synthetic oligonucleotides are purified by polyacrylamide gel electrophoresis (PAGE) or high-performance liquid chromatography (HPLC), their actual length in the preparation may vary due to the presence of deletions (more often) and insertions (less often) (Figure 4B). Second, our experience shows that most errors found in the reads come from imperfection in oligonucleotide primer synthesis and purification (however, this could strongly depend on a supplier). Therefore, substitutions, deletions, and insertions are quite possible in the sequences of the ROIs and BCs as well as in the regions of the constant parts flanking them (that were generated by oligonucleotides used at the plasmid library cloning step). The same is true for the edges of PCR-amplified products, which are introduced by appropriate primer pairs. Along with the general drop in the quality of sequencing toward the end of the reads, this is the main reason why we allow a fairly high percentage of errors (∼10%) in all expected parts of the reads. The described issues with the use of synthesized oligonucleotides are generally consistent with previous studies (Faircloth and Glenn, 2012; Hawkins et al., 2018). In addition, considering the possible variation in the BC length, especially its shortening (Figure 4B), it seems reasonable to equip the reference constructs that can be spiked into an MPRA library with slightly longer BC sequences (e.g., 20 nts in the example MPRA library). This could minimize the chance of accidental coincidence of sequences of the reference BC and a random BC.

Because many of the pipeline settings are arbitrary (set in the configuration file), it is important to note the following. First, of course, it is possible to set the allowed error level for all expected parts of reads to 0%; however, in the case of the example data set, this leads to a decrease in the number of genuine BCs by more than two times compared with the default settings described above. Second, because it is well known that the quality of sequencing gradually decreases toward the end of the reads, it seems appropriate to map the mCP3 and neCP2 regions in the reads not completely, but only by their beginnings. In particular, the use of only 10 instead of 17 nts for mCP3 and 20 instead of 86 nts for neCP2 for the example data set ultimately makes it possible to detect more than ∼1.5 times more genuine BCs with the error level in all parts of the reads set to 0%, but this gives only negligible gain (<0.1%) with the default settings described above. Third, the difference in the number of minimum reads, in which unique BCs should be detected in replicates of the mapping and normalization samples (parameters *m* and *n*), is associated with the fact that, when performing the mapping procedure, it is more important to identify the fact of different BC–ROI association(s) although data from the normalization samples are eventually quantified. Moreover, both of these parameters, as well as the parameter *e*, which determines the minimum number of reads for each unique BC in replicates of the expression samples, largely depend on both the complexity of a particular MPRA library (the number of unique clones in it) and the sequencing depth of the samples. Fourth, the threshold level of 0.9 controlling the identification of genuine BCs can be increased if necessary. This parameter is also highly dependent on the expected number of unique BC–ROI associations in the samples and their sequencing depth.

Although it is strongly recommended to obtain at least two biological replicates of the mapping, normalization, and expression samples, we notice that the pipeline nevertheless can process single replicates of these samples as well. This option can be useful when performing pilot experiments for a quick and preliminary evaluation of the results. Also, it is possible to load raw data obtained for different MPRA libraries into the pipeline simultaneously.

Finally, the results obtained for the example data set (Figure 4C) indicate that sequence variations in the region located after the TTS (which is not present in mature mRNA molecules) are able to substantially influence the reporter transcript level. This suggests a potentially high regulatory potential of the sequences located at the 3′-ends of genes, which has not yet been systematically studied.

## Data Availability Statement

The MPRAcode code written in Python is publicly available at https://github.com/Code-master2020/MPRAdecoder. The example input data as well as expected outputs are included in the GitHub repository. Detailed information on program can be found in the GitHub repository.

## Author Contributions

AL and AP conceived the study. AL, EO, and AI developed the pipeline. EO and AL performed experiments and applied the pipeline to the obtained data sets. AP supervised the project. AP, EO, and AL wrote the manuscript. All authors contributed to the article and approved the submitted version.

## Conflict of Interest

The authors declare that the research was conducted in the absence of any commercial or financial relationships that could be construed as a potential conflict of interest.

## Footnotes

1
https://github.com/Code-master2020/MPRAdecoder
