# Supplementary material for: MPRAdecoder: Processing of the Raw MPRA Data With a priori Unknown Sequences of the Region of Interest and Associated Barcodes
Source: Front Genet. 2021 May 11;12:618189. doi: 10.3389/fgene.2021.618189 (PMC8148044; doi:10.3389/fgene.2021.618189)
Supplement: Supplementary file 1 [file Image_1.PDF]

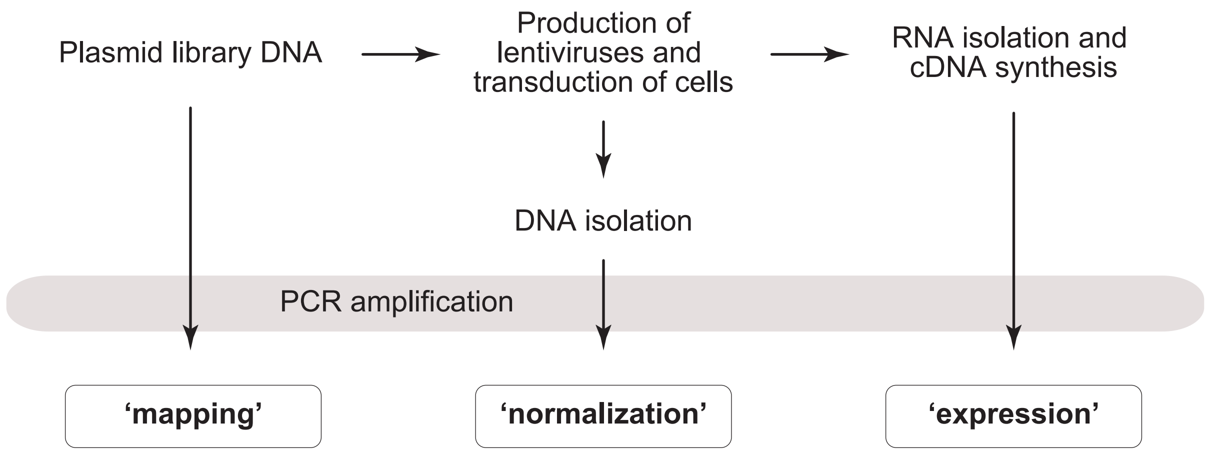

Supplementary Figure 1. Experimental steps involved in the preparation of the 'mapping', 'normalization', and 'expression' samples, in the case when MPRA constructs are randomly integrated into the target genome using lentiviruses.
